# Distinguishing glutamic acid in foodstuffs and monosodium glutamate used as seasoning by stable carbon and nitrogen isotope ratios

**DOI:** 10.1016/j.heliyon.2018.e00800

**Published:** 2018-09-19

**Authors:** Kazuhiro Kobayashi, Masaharu Tanaka, Soichi Tanabe, Yoichi Yatsukawa, Mitsuru Tanaka, Takuya Suzuki

**Affiliations:** a Global Food Safety Institute, Nissin Foods Holdings Co., Ltd., 2100 Tobuki-machi, Hachioji, Tokyo 192-0001, Japan; b Global Innovation Research Center, Nissin Foods Holdings Co., Ltd., 2100 Tobuki-machi, Hachioji, Tokyo 192-0001, Japan; c Graduate School of Biosphere Science, Hiroshima Univ. Kagamiyama, Higashihiroshima, Hiroshima 739-8528, Japan

**Keywords:** Food analysis

## Abstract

Recently, a number of consumers have begun to appreciate more natural ingredients and have become less willing to consume monosodium glutamate (MSG) as a seasoning. By measuring stable isotope ratios (δ^13^C and δ^15^N) of glutamic acid contained in foodstuffs and MSG used as seasoning, we attempted to distinguish between both using elemental analyzer-isotope-ratio mass spectrometry (EA/IRMS) and gas chromatography/combustion/IRMS (GC/C/IRMS). As a result, seasoning MSG was observed to have a lower δ^15^N value than glutamic acid in foodstuffs. We statistically analyzed the stable isotope ratio data using canonical discriminant analysis, thereby differentiating seasoning MSG from foodstuff-derived glutamic acid at an accuracy of 96.7%. This method is effective for distinguishing glutamic acid in foodstuffs from seasoning MSG.

## 1 Introduction

Umami seasonings may be divided into amino acid and nucleic acid types. Monosodium glutamate (MSG), an amino acid type of umami seasoning, is a sodium salt of glutamic acid and is consumed worldwide. However, a number of consumers have recently come to appreciate more natural ingredients and are less willing to consume MSG as a seasoning (Bandara et al., 2016), but distinguishing ingredients or the origins of foods based on product appearance alone is usually difficult. One of the analytical method for such discrimination is stable isotopic ratio analysis.

Isotope-ratio mass spectrometry (IRMS) has often been used in ecology and the geosciences to precisely measure stable isotope ratios of hydrogen, carbon, nitrogen, oxygen, and sulfur (Chikaraishi et al., 2009; Lawrence and Gedzelman, 1996). The stable isotopic ratios of these elements are also useful for determining the origin and authenticity of foodstuffs (Manca et al., 2001; Padovan et al., 2003; Suzuki et al., 2008; Raco et al., 2015). This approach is based on the principle that isotope ratios in food primarily reflect those either fixed or consumed by the organism from which the product is derived. In plants, the isotopic compositions of hydrogen and oxygen strongly reflect those ratios in the water absorbed by the plant, whereas the isotopic compositions of nitrogen and carbon reflect those of nitrogenous compounds in the soil and of carbon dioxide fixed through photosynthesis, respectively (Hoefs, 1996).

Glutamic acid plays an important role in amino acid metabolism of organisms (Forde and Lea, 2007). Glutamic acid is formed from glutamine and 2-oxoglutaric acid by glutamate synthase. The α-amino group of glutamic acid is transferred to other amino acids by the action of aminotransferase. In addition, the carbon skeleton of glutamic acid is the basis for the synthesis of γ-aminobutyric acid, arginine, and proline. Glutamic acid is deaminated by glutamate dehydrogenase to form ammonia and 2-oxoglutaric acid. Due to these reasons, it is considered that glutamate is abundant in common food sources, and previously glutamic acid was produced by extraction method (Royal, 1945).

Currently, MSG is generally produced by enzymatic microbial fermentation (Kinoshita et al., 1957; Kumagai, 2006). As a carbon source, sugarcane and sugar beet molasses, corn, cassava, and tapioca starch are used for MSG production, and ammonia or a salt thereof is used as a nitrogen source. When glutamic acid-producing bacteria (*Corynebacterium glutamicum*) are added and stirred, the fermentation produces glutamic acid. After recovering the produced glutamic acid by crystallization under acidic conditions, it is purified, neutralized with sodium hydroxide and decolorized to obtain the MSG crystal product (Saito et al., 2003).

The carbon isotopic composition (δ^13^C) of C4 plants, such as sugarcane and corn, significantly differs from that of C3 plants, including most crops, such as rice, wheat, cassava, tapioca, and sugar beet (C4: −16‰ to −9‰ vs. C3: −34‰ to −24‰) (Deines, 1980), because they utilize different photosynthetic (carbon fixation) pathways and grow in varied environments. Therefore, it should be possible to differentiate the origin of specimens based on differences in the isotopic compositions.

In this study, we attempted to distinguish the glutamic acid in foodstuffs and seasoning MSG by measuring the stable isotope ratios in glutamic acid. Glutamic acid is comprised of four elements: hydrogen, carbon, nitrogen, and oxygen. Of these elements, we chose to use a combination of δ^13^C and δ^15^N, which should directly reflect the isotope ratios of the substances absorbed by the livestock and crops from which the glutamic acid is derived. Given the potential differences that occur because of geographical factors, δD and δ^18^O were excluded as measurement targets. Recently, we have developed a method to isolate glutamic acid from various foodstuffs for precise determination of their δ^13^C (Kobayashi et al., 2018). We employed different pretreatment methods to measure the δ^13^C and δ^15^N values of glutamic acid. In this report, we observe that the glutamic acid contained in foodstuffs and seasoning MSG were distinguished well by performing canonical discriminant analysis to statistically analyze the obtained data.

## 2 Materials and methods

### 2.1 Samples

Table 1 shows the samples used in this study. Seasoning MSG products made in the United States, Japan, China, India, Thailand, Vietnam, Mexico, Brazil, and Hungary were obtained. For foodstuffs including protein hydrolysates, commercially available products purchased in the United States and Tokyo were used. All of the samples were purchased between 2015 and 2017. The samples were chosen as representatives of foodstuffs containing high level glutamic acid and consumed in Western countries or Japan.

**Table 1:** ^13^C and δ^15^N of glutamic acid in foodstuff and seasoning MSG. All isotopic data are averages of repeated measurements (n = 3). The HVP (hydrolyzed vegetable protein) and HAP (hydrolyzed animal protein) samples were hydrolyzed in advance.

Group	Sample	Country of purchase	δ^13^C (‰)	δ^15^N (‰)
C3 plants	Soy bean No. 1 (HVP)	Japan	−24.1	−0.8
	Soy bean No. 2 (HVP)	Japan	−22.9	2.0
	Soy bean No. 3 (HVP)	Japan	−24.0	−0.1
	Soy bean No. 4 (HVP)	the United States	−21.5	1.0
	Soy bean No. 5	Japan	−25.2	−1.0
	Tomato No. 1	Japan	−27.3	6.4
	Tomato No. 2	Japan	−27.2	−1.2
	Tomato No. 3	Japan	−26.8	4.6
	Potato, beet No. 1 (HVP)	Japan	−26.0	1.5
	Potato, beet No. 2 (HVP)	Japan	−26.0	0.5
	Wheat flour No. 1 (HVP)	Japan	−23.4	2.4
	Wheat flour No. 2	Japan	−25.2	0.5
	Broccoli	Japan	−26.6	2.1
	Chinese cabbage	Japan	−23.6	−2.4
	Soy bean, wheat flour (HVP)	the United States	−25.5	−1.0
	Spinach	Japan	−23.5	5.0
	Tea leaf	Japan	−22.7	0.5
C4 plants	Corn No. 1 (HVP)	Japan	−12.7	−2.8
	Corn No. 2 (HVP)	Japan	−13.1	−3.2
	Corn No. 3 (HVP)	Japan	−12.6	−0.2
	Corn No. 4 (HVP)	Japan	−13.5	3.7
	Corn No. 5 (HVP)	Japan	−13.0	2.2
	Corn No. 6 (HVP)	the United States	−14.0	−2.2
	Corn No. 7	Japan	−10.5	1.7
	Corn No. 8	Japan	−10.9	−3.1
Kelp	Makombu (*Laminaria Japonica*) No. 1	Japan	−10.3	4.8
	Makombu (*Laminaria Japonica*) No. 2	Japan	−12.6	5.9
	Rausu kombu (*Laminaria diabolica*) No. 1	Japan	−10.0	8.8
	Rausu kombu (*Laminaria diabolica*) No. 2	Japan	−11.1	7.7
	Rishiri kombu (*Laminaria ochotensis*) No. 1	Japan	−9.6	8.0
	Rishiri kombu (*Laminaria ochotensis*) No. 2	Japan	−10.4	4.0
	Hidaka kombu (*Laminaria angustata*)	Japan	−12.0	8.3
Marine products	Argentine red shrimp (*Pleoticus muelleri*)	Japan	−14.1	26.4
	Sakura shrimp (*Lucensosergia lucens*)	Japan	−14.0	20.0
	Whiteleg shrimp (*Litopenaeus vannamei*)	Japan	−16.8	14.6
	Big-eyed tuna (*Thunnus obesus*)	Japan	−14.0	29.0
	Pacific flying squid (*Tdoradodes pacificus*)	Japan	−16.2	22.1
	Sardine	Japan	−14.6	12.6
	Sardine, skip jack, tuna (HAP)	Japan	−12.4	19.7
	Scallop	Japan	−16.7	11.2
	Skip jack (HAP)	Japan	−12.1	23.5
	Yellow tail	Japan	−15.3	23.8
Mushrooms	Brown mushroom (*Agaricus bisporus*)	Japan	−19.5	17.1
	Elingi mushroom (*Pleurotus eryngii*)	Japan	−19.5	2.5
	Shiitake mushroom (*Lentinula edodes*)	Japan	−22.6	2.8
	Shimeji mushroom (*Hypsizygus marmoreus*)	Japan	−19.1	2.9
Livestock products	Beef	Japan	−14.6	14.9
	Chicken	Japan	−13.1	1.3
	Lamb meat	Japan	−24.4	14.3
	Pork	Japan	−13.0	1.6
	Cheddar cheese	Japan	−12.3	8.3
	Gouda cheese	Japan	−16.2	9.6
	Processed cheese	Japan	−18.1	5.2
MSG (C3 plants)	MSG No. 1	Hungary	−28.5	−2.7
	MSG No. 2	India	−29.1	−4.8
	MSG No. 3	India	−28.7	−5.3
	MSG No. 4	Thailand	−26.1	−6.6
	MSG No. 5	Thailand	−29.0	−6.1
	MSG No. 6	Vietnam	−26.2	−2.5
MSG (C4 plants)	MSG No. 7	Brazil	−13.3	−6.3
	MSG No. 8	Brazil	−12.4	−6.6
	MSG No. 9	China	−11.7	−8.8
	MSG No. 10	China	−12.3	−7.4
	MSG No. 11	China	−12.3	−8.4
	MSG No. 12	China	−12.5	−8.3
	MSG No. 13	China	−12.4	−6.5
	MSG No. 14	China	−12.8	−5.6
	MSG No. 15	China	−12.3	−9.3
	MSG No. 16	China	−11.8	−9.9
	MSG No. 17	China	−12.0	−6.3
	MSG No. 18	Japan	−15.6	−4.2
	MSG No. 19	Japan	−16.0	−3.9
	MSG No. 20	Japan	−10.9	−9.6
	MSG No. 21	Japan	−10.1	−9.3
	MSG No. 22	Japan	−10.7	−9.0
	MSG No. 23	Japan	−15.0	−6.3
	MSG No. 24	Japan	−15.2	−6.0
	MSG No. 25	Japan	−13.3	−6.6
	MSG No. 26	Japan	−14.7	−4.6
	MSG No. 27	Mexico	−11.3	−8.7
	MSG No. 28	Thailand	−12.7	−8.1
	MSG No. 29	Thailand	−12.3	−9.4
	MSG No. 30	Thailand	−11.6	−7.8
	MSG No. 31	Thailand	−12.1	−7.1
	MSG No. 32	the United States	−15.3	−5.1
	MSG No. 33	the United States	−14.1	−7.1
	MSG No. 34	Vietnam	−16.7	−6.0
	MSG No. 35	Vietnam	−14.8	−6.2
	MSG No. 36	Vietnam	−15.1	−4.9
	MSG No. 37	Vietnam	−15.0	−4.8

### 2.2 Reagents and chemicals

We purchased L-alanine (δ^13^C = −19.6‰, δ^15^N = 26.1‰), two types of glycine (δ^13^C = −32.3‰, δ^15^N = 1.12‰; δ^13^C = −60.0‰, δ^15^N = −26.6‰), L-histidine (δ^13^C = −11.4‰, δ^15^N = −7.6‰), L-hydroxyproline (δ^13^C = −12.7‰, δ^15^N = −9.2‰), L-leucine (δ^13^C = −28.4‰, δ^15^N = 6.2‰), and L-phenylalanine (δ^13^C = −11.2‰, δ^15^N = 1.7‰) from Shoko Scientific Corporation (Saitama, Japan) and used them as laboratory standards for the δ^13^C and δ^15^N analyses.

MSG monohydrate (MSG reagent, special grade), 25 vol% ammonia water (special grade), distilled water (HPLC grade), methanol (HPLC grade), dichloromethane (HPLC grade), 35.0–37.0 vol% hydrochloric acid (precision analysis grade), hexane (polychlorinated biphenyl testing grade), ammonium hydrogen carbonate (first grade), activated carbon (chromatograph grade), thionyl chloride, pivaloyl chloride, and anhydrous magnesium sulfate were purchased from Wako Pure Chemical Industries (Osaka, Japan). ODS-A-HG (particle diameter: 50 μm, micropore diameter: 12 nm; YMC, Kyoto, Japan) was used as the C18 resin. Amberlite® IR120 (H^+^ form; Dow Chemical, Midland, MI, US) and AG50W-X8 (H^+^ form, 200–400 mesh; Bio-Rad Laboratories, Tokyo, Japan) were used as the strong cation exchange resins, and Millex-LH (hydrophilic PTFE, pore diameter: 0.45 μm; Merck Millipore, Darmstadt, Germany) and Ultrafree-MC-GV (made of PTFE, pore diameter: 0.22 μm; Merck Millipore) were used for filtration.

### 2.3 Isolation of glutamic acid for δ^13^C

This process was performed according to our preliminary optimization study (Kobayashi et al., 2018) to isolate glutamic acid for carbon isotope ratio analysis. A total of 20 mL hydrochloric acid was added to the vegetables, beans, and mushrooms samples (6 g) and to the kelp, marine products, and livestock products samples (3 g) followed by heating at 110 °C for 20 h under a nitrogen atmosphere to hydrolyze proteins. After cooling, each sample was centrifuged at 2,140 × *g* for 3 min, and the supernatant was concentrated and dried with a rotary evaporator at 60 °C. The sample was dissolved in 30 mL of distilled water. Next, 6 mL of 1 M hydrochloric acid and 5 mL *n*-hexane/dichloromethane (3:2, v/v) were added, and the solution was stirred for 30 min. Following centrifugation at 2,140 × *g* for 3 min, the supernatant was removed and loaded onto an activated carbon (3 g)/C18 (3 g) column preconditioned with 30 mL of first methanol and later distilled water. After loading the sample, the column was eluted with 10 mL of distilled water. The effluent was collected and loaded onto a strong cation exchange (100 g) column preconditioned with 300 mL each of distilled water, 1 M hydrochloric acid, and distilled water again. After washing the loaded column with 400 mL of distilled water, the sample was eluted with 200 mL of 10 vol% ammonia water, and the eluate was concentrated and dried with a rotary evaporator at 60 °C. The residue was dissolved in 3 mL of 65 vol% methanol, filtered, and subsequently loaded in four approximately equal volumes of 0.1–0.5 mL to ensure sufficient peak separation onto the preparative high-performance liquid chromatograph (HPLC) column.

Glutamic acid was eluted for approximately 27 min using the conditions described below. The entire glutamic acid peak was carefully collected and dried with a rotary evaporator at 60 °C. The obtained glutamic acid crystals were wrapped in tinfoil and subjected to EA/IRMS to measure the δ^13^C and δ^15^N values. Since the glutamic acid crystals contained ammonia, which was derived from the mobile phase of HPLC and was difficult to remove, and the crystals showed an abnormal δ^15^N value, we measured the δ^15^N value by GC/C/IRMS. Seasoning MSG samples were not subjected to isolation or derivatization; instead, the samples underwent EA/IRMS as a raw powder to measure simultaneously both the δ^13^C and δ^15^N values.

### 2.4 Extraction and derivatization for δ^15^N analysis with GC/C/IRMS

This process was performed according to a previously reported method (Chikaraishi et al., 2007) to extract and derivatize glutamic acid for nitrogen isotope ratio analysis. Portions of the samples, equivalent to 0.2–0.5 mg of glutamic acid content, were collected. Each collected sample was mixed with 0.7 mL of 12 M hydrochloric acid, and the mixture was left to react at 110 °C for 12–24 h. Following filtration by centrifugation (Ultrafree-MC-GV, 40,000 × *g*, 1 min), the solution was sprayed with nitrogen at 110 °C for concentration and drying. After adding 0.6 mL of distilled water, 0.2 mL of 1 M hydrochloric acid, 0.3 mL of n-hexane, and 0.3 mL of dichloromethane, the solution was stirred and later centrifuged at 40,000 × *g* for 3 min. After removing the organic layer, the supernatant was loaded onto a strong cation exchange column (AG50W-X8, 10 mL) preconditioned by alternately flowing 15 mL of distilled water and 15 mL of either 1 M hydrochloric acid or 1 M sodium hydroxide. After washing with 40 mL of distilled water, the eluate was collected with 20 mL of 10 vol% ammonia water. Following vacuum concentration at 60 °C, the eluate was concentrated under a stream of nitrogen for drying.

The residue was mixed with 0.5 mL of thionyl chloride/isopropanol (4:1, v/v), and the mixture was left to react at 110 °C for 2 h. After the solution was concentrated under a stream of nitrogen for drying, the residue was added to 0.5 mL of pivaloyl chloride/dichloromethane (1:4, v/v), and the mixture was left to react at 110 °C for 2 h. After the reaction solution was concentrated under a stream of nitrogen for drying, 0.5 mL of n-hexane/dichloromethane (3:2, v/v) was added for extraction. The extracted solution was centrifuged using a filter (Ultrafree-MC-GV, 10,000 × g, 10 sec) filled with anhydrous magnesium sulfate. This procedure was repeated thrice. The solution was concentrated under a stream of nitrogen for drying and was later dissolved in 0.5 mL of dichloromethane. The resulting sample was subjected to GC/C/IRMS analysis. Similar to the δ^13^C measurements, the acid hydrolysis procedure was skipped during pretreatment of protein hydrolysates, and only the procedures from the defatting stage onward were performed.

### 2.5 Preparative HPLC

For isolation of glutamic acid, we used an HPLC system (Shimadzu Corporation, Kyoto, Japan) consisting of a controller (CBM-20A), liquid delivery pump (LC-20AP), auto-sampler (SIL-10AP), UV detector (SPD-20A), and fraction collector (FRC-10A). For preparative isolation, the column used was a Shodex Asahipak NH2P-90 20F (20 × 300 mm, particle size: 9 μm; Showa Denko, Tokyo, Japan), and the guard column used was a Shodex Asahipak NH2P-130G 7B (7.5 × 50 mm, particle size: 13 μm). The mobile phase was a mixture of 100 mM ammonium hydrogen carbonate and methanol (35:65, v/v) delivered at a flow rate of 7 mL min^−1^. The column temperature was set at room temperature, and ultraviolet absorbance at 210 nm was used for detection of compounds identified by their retention time.

The purity of the isolated glutamic acid crystals (ammonium salt) was verified using an HPLC system (Oji Scientific Instruments, Hyogo, Japan) consisting of a biosensor (enzyme electrode; BF-5) and an auto-sampler (BF-96AS). The glutamic acid crystals (20 mg) were dissolved in 100 mL distilled water prior to analysis.

### 2.6 EA/IRMS

Carbon isotope ratios (and nitrogen isotope ratios of seasoning MSG) were determined using an on-line system (Thermo Fisher Scientific, Bremen, Germany) comprised of a Delta V Advantage isotope-ratio mass spectrometer coupled to a Flash 2000 electron analyzer (EA) through a ConFlo IV interface. For elemental analysis, the combustion furnace used was a quartz glass tube (20 × 450 mm) filled with chromic oxide (100 mm) and silver-cobalt oxide (50 mm). The reduction furnace was a quartz glass tube (20 × 450 mm) filled with reduced copper (350 mm). All reagents were purchased from Thermo Fisher Scientific.

The combustion furnace, reduction furnace, and column oven of EA were set at 1,000 °C, 680 °C, and 50 °C, respectively. The flow rates of helium and oxygen were set at 100 and 175 mL min^−1^, respectively. Approximately 0.3 mg each of the standard reagents and 0.5 mg of each sample were weighed on a scale in tin capsules, and their carbon isotopic compositions were measured.

### 2.7 GC/C/IRMS

To measure nitrogen isotope ratios, we used an on-line system (Thermo Fisher Scientific) of a Delta V Advantage IRMS coupled to a GC instrument (Trace 1310) and GC Isolink II through a ConFlo IV interface. The reactor used for the GC instrument was comprised of a ceramic tube in which nickel and copper wires were inserted (Thermo Fisher Scientific). Ultra2 (50 m × 0.32 mm i.d., 0.52 μm film thickness, Agilent Technologies, Santa Clara, CA, US) was used as the capillary column.

The measurement conditions for GC were set according to previously reported methods (Chikaraishi et al., 2007; Takano et al., 2010). The column temperature was initially 40 °C (2.5 min hold) and was ramped at 20 °C min^−1^ to 110 °C (0 min hold), at 3.2 °C min^−1^ to 150 °C (0 min hold), 9 °C min^−1^ to 220 °C (10.0 min hold) and 30 °C min^−1^ to 250 °C (5.0 min hold). The temperature at the inlet was 270 °C, the injection dose was 1 μL (split-less), the flow rate was 1.4 mL min^−1^, and the temperature in the reactor was 1,000 °C.

From each of the standard reagents [as described in section 2.1 for L-alanine, glycine (δ^13^C = −60.0‰, δ^15^N = −26.6‰), L-hydroxyproline, L-leucine, and L-phenylalanine], 1 mg was collected and mixed. After derivatization using the method described in Section 2.4, the reagents were measured.

### 2.8 Isotope ratios

Stable isotope ratios (relative difference in stable isotope ratios between the standard specimen and sample, and represented as parts per thousand) were calculated via the following equation (Gonfiantini et al., 1995):

$$\delta^{\text{i}}\text{E(}‰\text{)}={\text{(R}}_{\text{sample}}{\text{/R}}_{\text{standard}}-1\text{)}\times 1000\text{,}$$

where i is the mass number of the heavier isotope of element E, R_sample_ signifies the stable isotope ratio of the sample (^13^C/^12^C or ^15^N/^14^N), and R_standard_ represents the isotope ratio of the international standard substance. For carbon, the international standard substance used was Vienna PeeDee Belemnite (VPDB). For nitrogen, the standard used was atmospheric nitrogen (AIR).

### 2.9 Canonical discriminant analysis

Based on the δ^13^C and δ^15^N values for glutamic acid in foodstuffs and seasoning MSG, the origins of which were already known, binomial canonical discriminant analysis was performed using the JMP 12.1.0 software (SAS Institute, Cary, NC, US) (Piasentier et al., 2003; Osorio et al., 2011) with quadratic fitting.

## 3 Results and discussion

### 3.1 δ^13^C and δ^15^N of glutamic acid in foodstuffs and seasoning MSG

Several reports have indicated that isotopic fractionation can be observed. When isotopic fractionation is observed, isotope ratios are altered during pretreatment procedures, such as purification and derivatization, prior to measuring stable isotope ratios (Macko et al., 1987; Silfer et al., 1991). Nevertheless, we previously confirmed that subjecting a reagent-grade MSG sample to hydrolysis by hydrochloric acid did not significantly change δ^13^C values (Kobayashi et al., 2018) in a series of pretreatment steps. Isotope carbon ratio values before and after hydrolysis step of the reagent MSG were −14.0 ± 0.1‰ and −14.1 ± 0.1‰, respectively. When a similar analysis testing the effect of the hydrolysis was performed for δ^15^N, the δ^15^N values of the reagent MSG were −6.0 ± 0.6‰ and −6.3 ± 0.1‰ with or without hydrolysis, respectively. Although the δ^13^C and δ^15^N values were measured using kelp as a foodstuff example, no significant variations in sample with or without hydrolysis step were observed (with hydrolysis δ^13^C −10.0 ± 0.5‰, δ^15^N 8.8 ± 0.9‰ and without hydrolysis δ^13^C −10.6 ± 0.3‰, δ^15^N 8.9 ± 1.1‰).

**Fig. 1:**
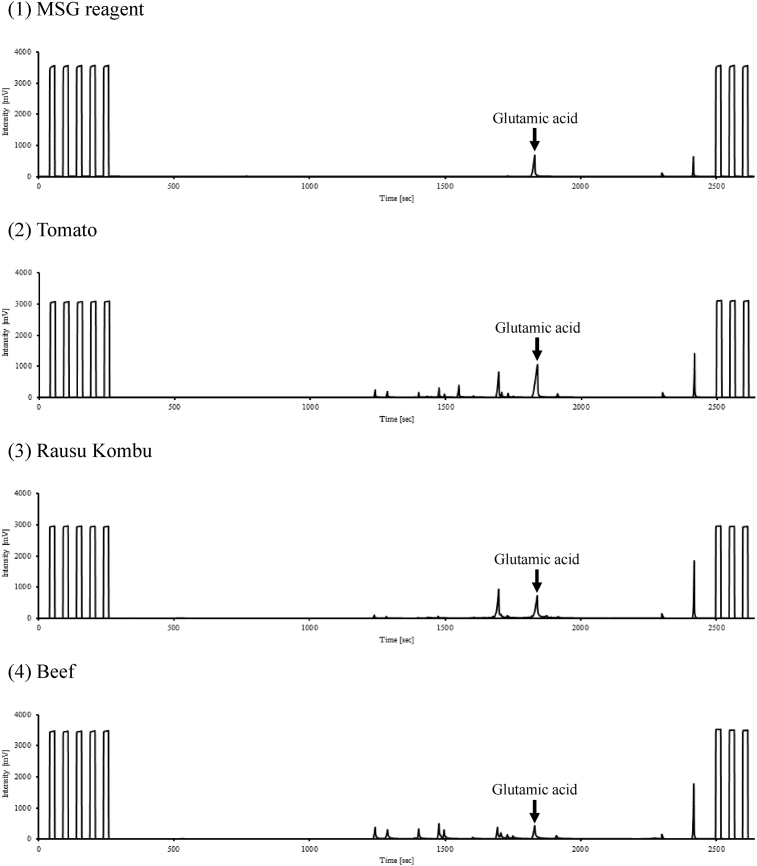
GC/C/IRMS chromatograms on nitrogen isotope analysis of amino acid solution extracted from MSG reagent and foodstuff samples: (1) MSG reagent, (2) Tomato, (3) Rausu kombu, and (4) Beef.

Fig. 1 shows the GC/C/IRMS chromatograms on nitrogen isotope analysis of amino acid solution extracted from MSG reagent and foodstuff samples. The peak of glutamic acid was separated clearly from the peaks of other amino acid.

**Fig. 2:**
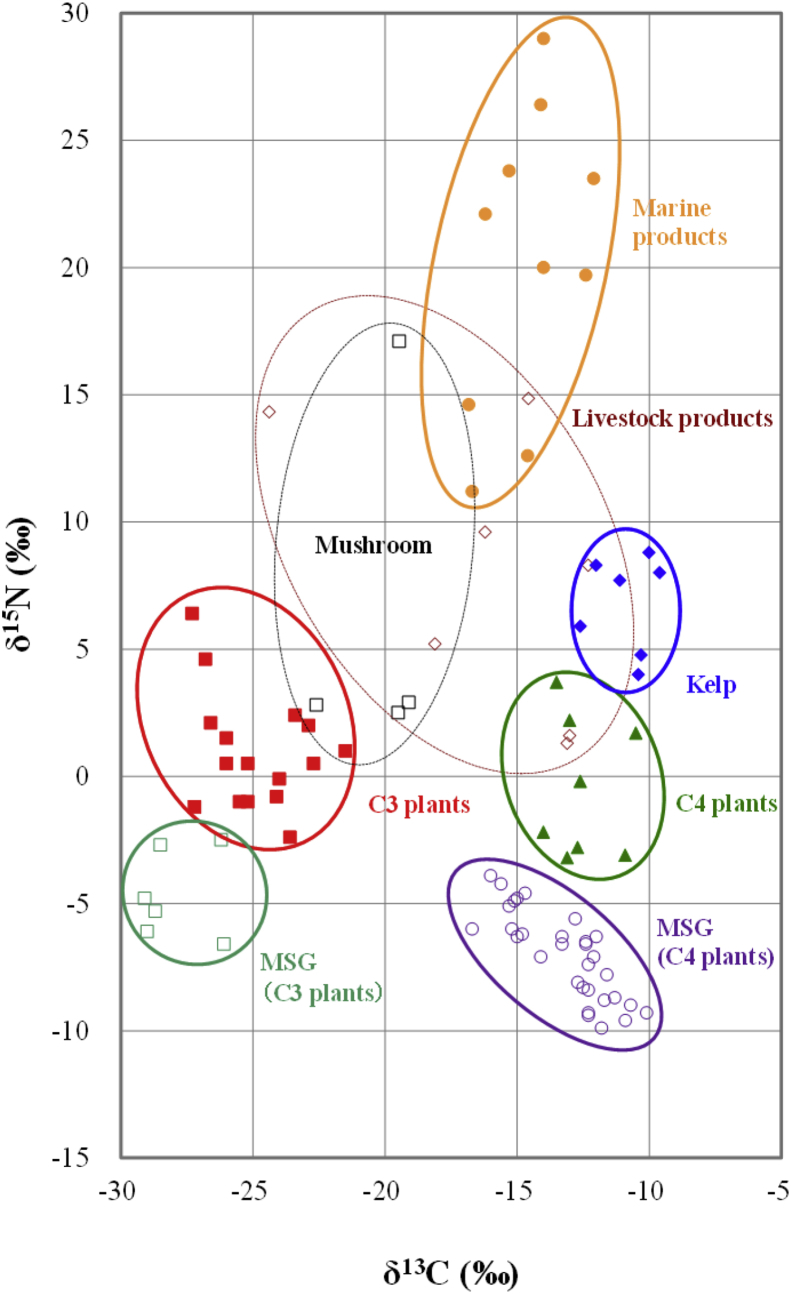
δ^13^C and δ^15^N value from glutamic acid in foodstuffs and seasoning MSG.

Fig. 2 and Table 1 show the results of the δ^13^C and δ^15^N measurements of glutamic acid in foodstuffs and seasoning MSG. The purity of the isolated glutamic acid for δ^13^C measurement was in the range of 96–100%. The δ^13^C values of glutamic acid excluding seasoning MSG were distributed between relatively high values (−18.1‰ to −9.6‰) and relatively low values (−27.3‰ to −21.5‰). The former group was further divided with respect to the δ^15^N values into corn (C4 plants: −3.2‰ to 3.7‰), kelp (4.0‰–8.8‰), and marine products (11.2‰–29.0‰). The latter group overlapped the range associated with vegetables/beans (C3 plants: −2.4‰ to 6.4‰). A probable reason for the slightly wider distribution of δ^15^N may be that nitrogenous compounds present in fertilizers (chemical fertilizers/organic manures) were applied to soil and affected the δ^15^N value (Bateman et al., 2007).

In ecological research, the δ^15^N values of the marine products are well-documented, with isotopic weights tending to increase as the product originates from a higher level in the food chain (Chikaraishi et al., 2007). In this study, the δ^15^N value of glutamic acid was 29.0‰ in big-eyed tuna, 23.8‰ in yellowtail, 23.5‰ in skipjack, 22.1‰ in Pacific flying squid, 12.6‰ in sardine, 11.2‰ in scallop, and 4.0‰ to 8.8‰ in kelp. The δ^15^N values were approximately in the order of the level in the food chain. However, there were some exceptions, such as 14.6‰ in whiteleg shrimp (cultivated) and 26.4‰ in Argentine red shrimp (wild).

Mushrooms (shiitake and common mushrooms) were partly different from other foodstuffs in terms of the δ^15^N and δ^13^C values of glutamic acid. Instead of photosynthesis, mushrooms depend on other organisms for nutrition. Consequently, the differences in the carbon and nitrogen sources may have a large impact on the isotope ratios. The carbon and nitrogen isotope ratios of glutamic acid in livestock products, including meat and cheese, were also widely distributed, possibly due to the diversity of the food items fed to livestock.

The δ^13^C values from the seasoning MSG samples exhibited two distinct distribution patterns in ranges of −29.1‰ to −26.1‰ and −16.7‰ to −10.1‰. The former range may be derived from C3 plants, including tapioca, cassava, and sugar beet, whereas the latter range may be derived from C4 plants, including sugarcane and corn (Deines, 1980). For the seasoning MSG samples, the δ^15^N values from the C3 plant-derived samples ranged between −6.6‰ and −2.5‰, whereas those from the C4 plant-derived samples ranged between −9.9‰ and −3.9‰.

In comparison with the δ^15^N values from glutamic acid in foodstuffs, those from seasoning MSG were slightly lower isotopically. When seasoning MSG is produced by enzymatic microbial fermentation, the common nitrogen source is usually ammonia gas in solution (Kinoshita et al., 1957; Kumagai, 2006). Because the isotopic ratio of gaseous nitrogen of the raw material of ammonia is 0‰, that of seasoning MSG is also expected to be 0‰. The reason why seasoning MSG exhibited slightly lower nitrogen isotope ratios was assumed to be the isotopic fractionation of nitrogen that occurs during the MSG purification processes in manufacture, such as decolorization and recrystallization (Saito et al., 2003).

### 3.2 Canonical discriminant analysis using δ^13^C and δ^15^N of glutamic acid in foodstuffs and seasoning MSG

A total of 90 samples with known origins were tested and divided into the following three groups: I) foodstuffs (53 samples), II) seasoning MSG C3 plants (6 samples), and III) seasoning MSG C4 plants (31 samples). Canonical discriminant analysis was performed on the isotopic data for the glutamic acid in these samples. The discriminant functions obtained are as follows:

Function 1: 0.154 [δ^15^N] +0.141 [δ^13^C] −2.744,
Function 2: 0.165 [δ^15^N] +0.073 [δ^13^C] +2.688.

**Fig. 3:**
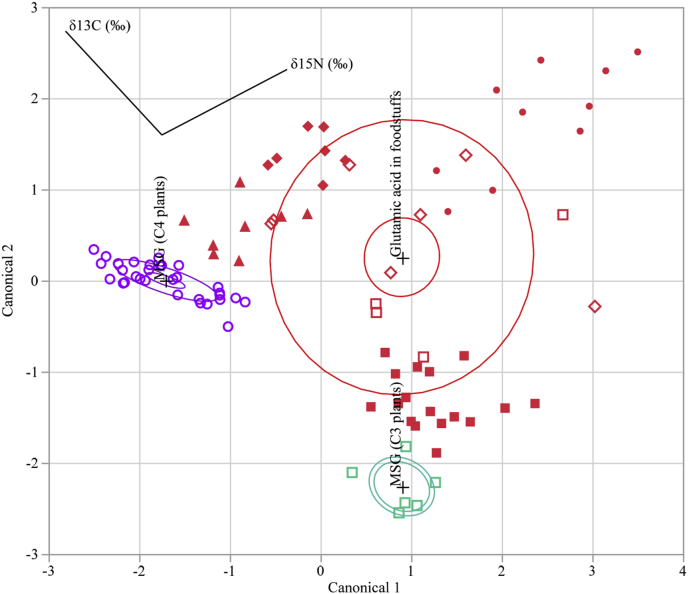
Canonical discriminant plot using δ^13^C and δ^15^N values from glutamic acid in foodstuffs and seasoning MSG. Eigenvalue, contribution, cumulative contribution and canonical correlations in Function 1 and 2 are shown. More details are provided in the results section.

Table 2 lists the eigenvalues, contributions, cumulative contributions, and canonical correlations obtained from the analysis. The function 1 and 2 eigenvalues were 1.587 (contribution: 80.2%) and 0.392 (contribution: 19.8%), respectively, showing that the function 1 variables contain significantly more information that can be used to distinguish the groups. The canonical correlation of function 1 with a high contribution rate was sufficiently high (0.783) for group differentiation. By substituting the measurement data of each sample in the discriminant functions, we calculated score points for the function 1 and 2 variables. Based on these score points, we created a scatter diagram with the function 1 and 2 score points plotted on the horizontal (canonical 1) and vertical axes (canonical 2), respectively (Fig. 3).

**Table 2:** Canonical discriminant analysis details using the δ^13^C and δ^15^N values from glutamic acid in foodstuffs and seasoning MSG. Eigenvalue, contribution, cumulative contribution and canonical correlations in Function 1 and 2 are shown. More details are provided in the results section.

	Function 1	Function 2
Eigenvalue	1.587	0.392
Contribution (%)	80.2	19.8
Cumulative contribution (%)	80.2	100.0
Canonical correlations	0.783	0.531

The inner ellipses represent a 95% confidence level for each mean, and the outer ellipses represent a 50% contour for each group, which is a region in the space of the two canonical variables that contains approximately 50% of the observations, assuming normality. The direction of a ray indicates the degree of association for a covariate with the two canonical variables. The coordinate points (canonical 1 and 2) corresponding to group means of the glutamic acid in foodstuffs, seasoning MSG (C3 plants) and seasoning MSG (C4 plants) are (0.898, 0.256), (0.897, −2.260), and (−1.709, 0.000), respectively, and are denoted by plus (“+”) signs.

Using this method, 87 of 90 samples (96.7%) were correctly differentiated, demonstrating that the method is effective for differentiating seasoning MSG from glutamic acid in foodstuffs.

On the other hand, the origins of glutamic acid in eight groups, vegetables/beans/crops (C3 and C4 plants), marine products (seafood and kelp), seasoning MSG (derived from either C3 or C4 plants), livestock products and mushrooms were differentiated at an accuracy of 90.0%. The reason for the slightly lower accuracy is likely due to the range of the stable isotope ratio of some livestock products and mushrooms overlapping the range of vegetables/beans/crops (C3 and C4 plants) and marine products (seafood and kelp). Differentiation of glutamic acid contained in these samples is a task that should be performed in the future.

## 4 Conclusions

By measuring the δ^13^C and δ^15^N values from samples of glutamic acid in foodstuffs and seasoning MSG, we attempted to distinguish them. The isotope ratio values for the carbon and nitrogen from the glutamic acid in foodstuffs were separately measured. Carbon isotope ratio values were measured using EA/IRMS after the glutamic acid was isolated according to the preliminary optimization of our pretreatment method (Kobayashi et al., 2018). Nitrogen isotope ratio values were measured using GC/C/IRMS according to a pretreatment method previously reported (Chikaraishi et al., 2007). For seasoning MSG samples, both the δ^13^C and δ^15^N values were measured using EA/IRMS.

Data obtained regarding the stable isotope ratios were statistically analyzed using canonical discriminant analysis to distinguish between glutamic acid in a wide variety of foodstuffs and seasoning MSG with an accuracy of 96.7%. Three samples of glutamic acid derived from C3 plants were erroneously judged to be seasoning MSG of C3 plant origin. It is considered that the difference between δ^15^N value of glutamic acid derived from C3 plants and seasoning MSG was closer than the difference between δ^15^N value of glutamic acid derived from C4 plants and seasoning MSG. Nevertheless, these results demonstrate that this method is effective for distinguishing glutamic acid in food samples and seasoning MSG.

## Declarations

### Author contribution statement

Kazuhiro Kobayashi: Performed the experiments; Wrote the paper.

Masaharu Tanaka, Yoichi Yatsukawa: Conceived and designed the experiments; Performed the experiments; Analyzed and interpreted the data; Contributed reagents, materials, analysis tools or data; Wrote the paper.

Soichi Tanabe, Mitsuru Tanaka, Takuya Suzuki: Analyzed and interpreted the data; Wrote the paper.

### Funding statement

This research did not receive any specific grant from funding agencies in the public, commercial, or not-for-profit sectors.

### Competing interest statement

The authors declare no conflict of interest.

### Additional information

No additional information is available for this paper.
